# Effect of health education combined with biofeedback electrical stimulation on early pelvic floor function and psychology: A retrospective study

**DOI:** 10.1097/MD.0000000000039321

**Published:** 2024-08-23

**Authors:** Feng Yuan, Ying Hu, Chunrong Yang

**Affiliations:** 1 Department of Gynecology and Obstetrics, Shaanxi Provincial People’s Hospital, Xian, China

**Keywords:** anxiety and depression, Greene health education, health education, Kegel pelvic floor rehabilitation training, pelvic floor muscle strength, quality of sexual life.

## Abstract

To investigate the effects of health education combined with biofeedback electrical stimulation on maternal postpartum pelvic floor function and psychology. The clinical data of 80 patients with postpartum pelvic floor dysfunction (PFD) from May 2020 to May 2022 were selected as retrospective study subjects, and 40 cases were divided into the comparison group and the observation group according to the different intervention methods. Among them, the comparison group implemented biofeedback electrical stimulation and guidance, and the observation group implemented Greene health education and Kegel pelvic floor rehabilitation training intervention based on the comparison group. The differences in pelvic floor muscle strength, sexual quality of life, anxiety, and depression in the 2 groups with postpartum PFD were compared. Comparison of pelvic floor muscle strength: before the intervention (*P* > .05) and after the intervention, the anterior resting mean electromyography (EMG), slow muscle mean EMG, fast muscle maximum EMG, and mixed muscle mean EMG values of patients in the observation group were higher than those in the comparison group, and the posterior resting mean EMG values were lower than those in the comparison group (*P* < .05). There was no statistically significant difference in the Hospital Anxiety and Depression Scale (HADS) scores and anxiety and depression subscale scores between the 2 groups of patients before intervention (*P* > .05). After the intervention, the HADS scores and anxiety and depression subscale scores were lower than those before the intervention in both groups, and the differences were statistically significant in the intervention group than in the comparison group (*P* < .05). There was no statistically significant difference between The Chinese Female Sexual Life Quality Questionnaire scores of both groups before the intervention (*P* > .05). Sexual desire, vaginal lubrication, sexual arousal, sexual satisfaction, orgasm, and painful intercourse improved in both groups after the intervention, and the scores in the intervention group were higher than those in the comparison group (*P* < .05). Health education combined with biofeedback electrical stimulation can effectively improve the quality of patients’ sexual life, improve the pelvic floor muscle strength of patients with postpartum PFD, enhance patients’ confidence, reduce patients’ anxiety and depression, and effectively improve patients’ psychological status.

## 1. Introduction

Pelvic floor dysfunction (PFD) is a disease caused by various causes of damage and defects in pelvic floor support structures leading to PFD and even pelvic organ displacement, and patients may suffer from sexual dysfunction, urinary incontinence, pelvic organ prolapse, and other symptoms, which seriously affect the quality of life.^[1]^ The incidence of female PFD in China is 40%, and female PFD is caused by congenital defects or injuries to the pelvic floor support structures, which mainly manifests clinically as pelvic organ prolapse, stress urinary incontinence, and female sexual dysfunction. The pathogenesis is mainly related to pregnancy and childbirth damage to the pelvic floor support structures.^[1]^ The pelvic floor function is also affected by age, aging will lead to a decline in pelvic floor function, and the above clinical manifestations will develop from mild to moderate to severe, which will seriously affect women’s daily life and work and lead to physical and psychological injuries.^[2]^

At present, treatment options for PFD include surgical and nonsurgical treatment, with nonsurgical treatment being the main treatment for mild patients and Kegel pelvic floor rehabilitation training being the main pelvic floor muscle training method, and Kegel pelvic floor muscle rehabilitation training can be used as an adjunctive therapy and postoperative rehabilitation for severe patients.^[3]^ With the improvement of living standards and the promotion of the postpartum rehabilitation concept, pelvic floor rehabilitation training has been gradually applied in early clinical rehabilitation, and studies have shown that it can effectively strengthen maternal pelvic floor muscle strength and improve sexual satisfaction and urinary control ability in the postpartum period.^[4]^ Although Kegel pelvic floor rehabilitation training is effective, patient compliance and motivation are difficult to ensure due to the lack of professional guidance and insufficient disease awareness.^[5]^ The Greene health education model is a multidisciplinary perspective and targeted health education and health intervention model.^[6]^ Biofeedback electrical stimulation is a method of instructing patients to obtain proper and effective pelvic floor training by directly stimulating the patient’s muscles and nerve tissues with electric current to awaken the patient’s proprioceptors and induce muscle training.^[7]^ Biofeedback electrical stimulation can improve pelvic floor muscle function by combining biofeedback electrical stimulation therapy with Greene health education combined with Kegel pelvic floor rehabilitation training.^[8]^ In our study, we compared Greene health education combined with Kegel pelvic floor rehabilitation training with conventional interventions to analyze their effects on pelvic floor muscle function, sexual dysfunction, and stress urinary incontinence in patients with postpartum PFD in order to understand their effects on promoting the recovery of postpartum pelvic floor function and improving patients’ quality of life.

## 2. Material and methods

### 2.1. Research object

This study was approved by the Ethics Committee of Shaanxi Provincial People’s Hospital. The clinical data of 80 patients with postpartum PFD from May 2020 to May 2022 were selected as the subjects of a retrospective study, and 40 cases were divided into a comparison group and an observation group according to the intervention method. PFD diagnosis^[4]^: defecography showed functional and organic lesions Q in the Q area of the anal canal and rectum, and anorectal palpation felt that a high level of tension in the muscles responsible for defecation in patients with Q muscle potentials was abnormal.

### 2.2. Inclusion and exclusion criteria

Inclusion criteria: singleton primigravida, full-term gestational delivery, diagnosis of PFD by postpartum pelvic floor muscle strength examination, clear consciousness, fluent expression, able to complete the treatment cycle, and age ≥18 years, with elementary school education or above; patients with self-care ability, able to understand and cooperate with our study, and detailed and complete clinical data of patients and their families; with symptoms of urinary leakage, all are normal gestational primigravida and the patients were able to understand and cooperate with our study; and they had symptoms of urinary leakage and were normal first-time mothers with normal pregnancies. Exclusion criteria: those who had PFD diseases, pelvic surgery, pelvic floor injury, chronic respiratory diseases, and constipation before pregnancy; those who had gestational diabetes mellitus, gestational hypertension, placenta praevia, amniotic fluid hypohydramnios (maximum dark area of amniotic fluid ≤2-cm vertical depth or amniotic fluid index ≤5-cm in late pregnancy), amniotic fluid hyperhydramnios (maximum dark area of amniotic fluid ≥8-cm vertical depth or amniotic fluid index ≥25 cm) during pregnancy (amniotic fluid index ≥25 cm), and other pathological conditions; those with a history of malignant tumors, newborns with low weight, giant babies, breech, and twin fetuses; those with other medical or surgical diseases combined with pregnancy; and those who cannot undergo rehabilitation training due to physical reasons, such as cervical spondylosis, bone and joint diseases, visual impairment, hemiplegia, and pregnancy.

### 2.3. Methods

In the comparison group, biofeedback electrical stimulation and guidance were implemented, that is, electrical stimulation treatment was first performed using a biostimulation feedback device. The patient was placed in a 30° supine position, the vaginal electrodes were placed in the vagina, the frequency was adjusted to 8 to 32 Hz, and the pulse width distribution was adjusted to 320 to 740 µs so that the tension and muscle strength of class I fibers were fully restored. Then, the frequency was adjusted to 20 to 80 Hz and the pulse width was adjusted to 20 to 320 s to fully restore the tone and muscle strength of class II fibers, 2 times/wk, 10 to 15 min/time, 10× per session, and 2 sessions in total. Subsequently, in the biofeedback mode, different training modules were selected, and patients were instructed to perform pelvic floor muscle group contraction training according to the electromyography (EMG) formed by the biostimulation feedback instrument, 2 times/wk, 15 to 20 min/time, 10× per session, and for a total of 2 sessions. Patients were instructed to go home and supplemented with pelvic floor muscle exercises, also known as Kegel training. The specific method is given as follows: the patient takes a flat lying position, bends the legs slightly apart, tries to contract the anus for 6 to 8 s when inhaling, relaxes when exhaling, repeats the above action at an interval of 5 to 10 s, and continuously does 15 to 30 minutes, 2 to 3 times/d, each course of treatment 4 to 6 weeks, and total 2 courses of treatment.

In the observation group, Green health education and Kegel pelvic floor rehabilitation training intervention were applied on the basis of the comparison group, that is, pelvic floor rehabilitation exercise: the patient emptied the bladder, took a supine, standing or sitting position, and performed pelvic floor muscle contraction under the guidance of medical professionals, that is, inspiratory efforts to contract the anus, vagina, and urethra, pay attention to the relaxation of abdominal and hip muscles during inspiration, and hold for ≥ 3s as much as possible, followed by slow exhalation to relax the muscles. The exercise should be repeated for 10 s, and the contraction time should be prolonged as much as possible after proficiency; each contraction should be ≥10 s, and the duration of 1 exercise should be ≥15 min, 3×/d, and 8 weeks as a course of treatment. Health education: in-depth and detailed interviews were conducted with the mothers, and the factors affecting PFD were categorized into 3 categories: propensity, facilitation, and reinforcement. Tendency factors: through meticulous communication, we found that patients and their families lacked a proper understanding of PFD and ignored its possible hazards, such as the probability of suffering from postpartum depression, urinary tract infection, vulvar infection, and urinary incontinence. Response: the department provides weekly health education lectures and training on disease-related knowledge before intervention, including the occurrence and development factors of PFD and treatment methods, helping patients to establish health beliefs, making them fully aware of the necessity of adhering to exercise and improving patient compliance. Contributing factors: at present, the treatment of PFD is divided into surgical treatment and nonsurgical treatment. Nonsurgical treatment is mainly pelvic floor muscle rehabilitation training, which has certain effects, but requires patients to exercise for a long time and correctly. In practice, patients are often unable to grasp the correct exercise method and adhere to the exercise due to misunderstanding, lack of memory, or lack of patience, which makes it difficult to achieve the desired exercise effect. The main contents were the diagnosis and treatment of PFD, the main points and common mistakes of Kegel pelvic floor rehabilitation training, the specific implementation steps of Green health education, etc. After a comprehensive assessment of the patients, a targeted self-management schedule was formulated, and 1 case of Kegel pelvic floor rehabilitation training was arranged for each hour in the afternoon of each day for health education. The nursing staff instructed the patient to put the index and middle fingers into the vagina and feel the sensation of wrapping the fingers around the pelvic floor muscles when they contracted, told the patient to keep contracting for ≥3 s, try to extend the contraction time, and relax for 10 s after the contraction was over. Exercise effect enhances patient confidence. Reinforcement factors: PFD can recover naturally to a certain extent, but as time goes by, the patient’s self-recovery ability decreases, even with the progressive development of age, and there may be slow effects or aggravation of the disease due to accidental factors, leading to the patient’s doubts about the effect of exercise and affecting treatment compliance. Response: establish a WeChat communication group, which includes medical staff, patients, and their families. Patients perform Kegel pelvic floor rehabilitation training daily according to the self-management schedule and self-evaluate the training effect, such as urine leakage, interval time between urination, frequency of sex life, satisfaction with sex life, and psychological condition. The nursing staff follows up on the patient’s training intensity daily, gives feedback and analysis, and answers questions on their learning progress and training effects; follows up on the patient’s weekly visits to the hospital, and understands the patient’s pelvic floor muscle strength recovery through biofeedback electrical stimulation therapy device and questionnaires; and encourage patient interaction, and encourage the patient’s family to give the patient more understanding, support and, play a supervisory role.

### 2.4. Observation indexes

Sexual life quality: The Chinese Female Sexual Life Quality Questionnaire 6 was used for evaluation, with a total of 19 entries, which can be divided into 6 dimensions: sexual desire (2 entries), orgasm (3 entries), vaginal lubrication (4 entries), sexual life satisfaction (3 entries), sexual arousal (4 entries), and painful intercourse (3 entries), with each entry scoring from 1 to 5. The higher the score, the higher the quality of sexual life. The Hospital Anxiety and Depression Scale (HADS) score is used to screen for anxiety and depression in patients attending general medical clinics and has the advantage of being simple, quick, and easy to use. It consists of 2 subscales that rate depression and anxiety, each with 7 entries, 4 answers per entry, and a score of 0 to 3. The scores of the 2 subscales of anxiety and depression are divided as follows: 0 to 7 for no symptoms, 8 to 10 for suspicious symptoms, and 11 to 21 for definitely existing symptoms. The MyoTrac Infiniti biostimulation feedback instrument manufactured by Nanjing Weiss was used to evaluate the recovery of pelvic floor muscles in both groups: the indexes included anterior resting mean EMG value, slow muscle mean EMG value, fast muscle maximum EMG value, mixed muscle mean EMG value, and posterior resting mean EMG value of pelvic floor muscles.

### 2.5. Statistical methods

All data of our study were checked using Excel double entry and SPSS 28.0 for statistical analysis, setting the test level α = 0.05 and considering *P* < .05 as a statistically significant difference. Statistical descriptions of measurement data obeyed normal distribution and were described by mean ± standard deviation, those not obeying normal distribution were described by median (interquartile spacing), and count data were described by frequency and composition ratio. General patient data were analyzed: categorical data were analyzed by χ^2^ test, continuity-corrected χ^2^ test, and Fisher exact probability method; measurement data were analyzed by the *t* test. With obedience to a normal distribution, paired samples *t* test was used for within-group comparisons and 2 independent samples *t* test for between-group comparisons; with disobedience to a normal distribution, a nonparametric Wilcoxon signed-rank test was used for within-group comparisons, and the rank-sum test was used for between-group comparisons for analysis.

## 3. Results

### 3.1. Baseline data comparison

There was no significant difference in the average age, gestational age, educational level, and weight of the patients in the observation group, and the difference was not statistically significant (*P* > .05; Table 1).

**Table 1:** Comparison of baseline data of 2 groups of patients.

Group	Average age, yr	Gestational age, wk	Weight, kg	Educational level		
				Junior high school	High school	University and above
Comparison group (40)	60.90 ± 1.71	39.52 ± 1.71	66.35 ± 2.10	12	12	16
Observation group (40)	61.10 ± 1.62	39.48 ± 1.63	64.10 ± 1.10	13	10	17
*t*	0.377	0.107	2.107	0.252		
*P*	.051	.915	.079	.882		

### 3.2. Comparison of pelvic floor muscle strength

**Figure 1.**
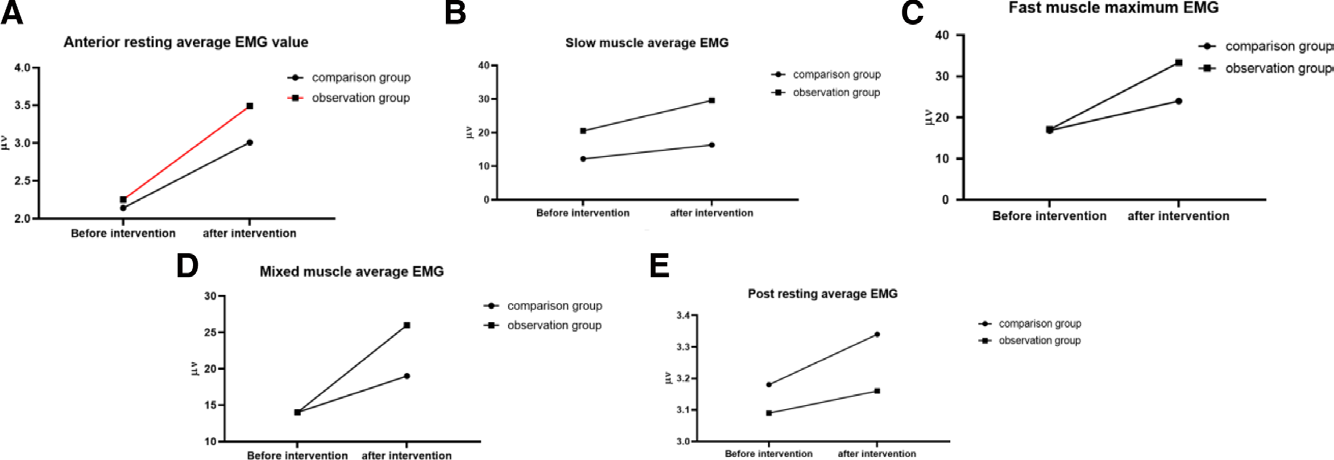
Comparison of pelvic floor muscle strength (in our study, all pelvic floor muscle strength data were checked by the double entry in Excel, and SPSS 28.0 was used for statistical analysis). The statistical descriptive measurement data obeyed the normal distribution and were described by the mean ± standard deviation, and those that did not obey the normal distribution were described by the median (four-quantile interval), which is used to describe the count data by frequency and composition ratio. Paired samples *t* test was used for intragroup comparison, and the 2 independent samples *t* test was used for intergroup comparison. It was found that there was no significant difference in pelvic floor muscle strength between the 2 groups before intervention (*P* > .05). The anterior resting average electromyography (EMG) value, slow muscle average EMG value, fast muscle maximum EMG value, and mixed muscle average EMG value of the pelvic floor muscles in the group were higher than those in the control group, and the posterior resting average EMG value was lower than the comparison group. The difference was statistically significant (*P* < .05).

Before the intervention, there was no statistically significant difference in pelvic floor muscle strength between the 2 groups (*P* > .05); after the intervention, the anterior resting mean EMG, slow muscle mean EMG, fast muscle maximum EMG, and mixed muscle mean EMG values of patients in the observation group were higher than those in the comparison group; and the posterior resting mean EMG values were lower than those in the comparison group, with statistically significant differences (*P* < .05; Fig. 1).

### 3.3. Comparison of anxiety and depression

**Figure 2.**
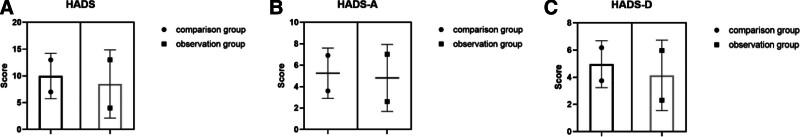
Comparison of pelvic floor muscle strength (in our study, all anxiety and depression data were checked by a double entry in Excel, and SPSS 28.0 was used for statistical analysis). The statistical descriptive measurement data obeyed the normal distribution and were described by the mean ± standard deviation, and the nonnormal distribution was described by the median (quartile). The count data are described by frequency and composition ratio. Paired samples *t* test was used for intragroup comparison, and the 2 independent samples *t* test was used for between-group comparison. It was found that there was no significant difference in the scores of Hospital Anxiety and Depression Scale (HADS) score and anxiety (HADS-A) and depression (HADS-D) subscale scores between the 2 groups before intervention (*P* > .05). After the intervention, the HADS score and HADS-A and HADS-D subscale scores of the 2 groups of patients were all lower than those before the intervention, the intervention group was lower than the control group, and the difference was statistically significant (*P* < .05).

There was no significant difference in the scores of HADS and anxiety and depression subscale scores between the 2 groups before intervention (*P* > .05). After the intervention, the HADS score and anxiety and depression subscale scores of the 2 groups of patients were all lower than those before the intervention, the intervention group was lower than the control group, and the difference was statistically significant (*P* < .05; Fig. 2).

### 3.4. Comparison of anxiety and depression

**Figure 3.**
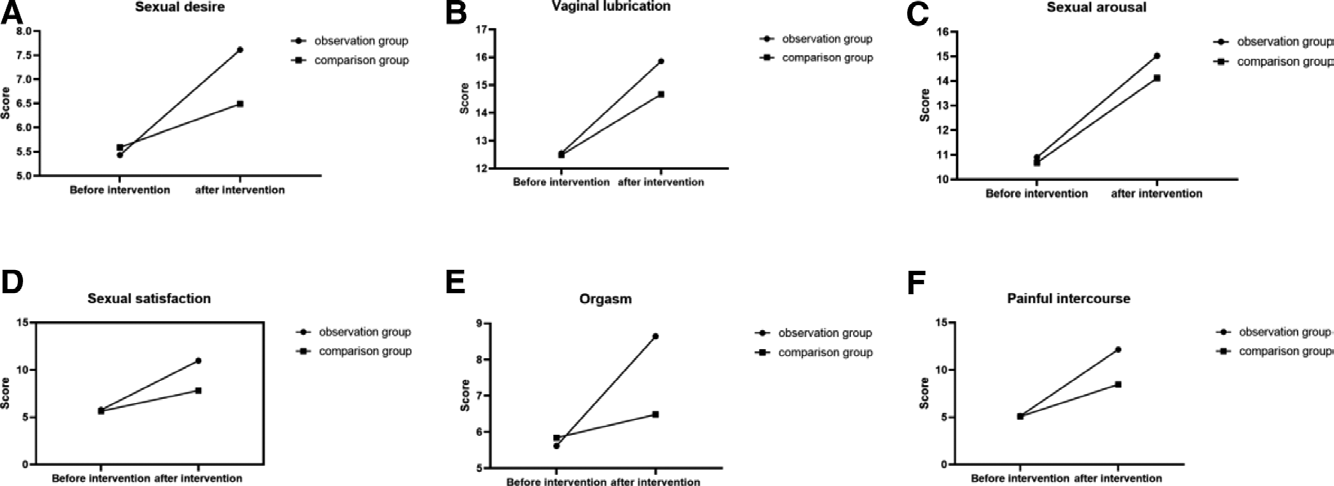
Comparison of anxiety and depression (in our study, all sexual life quality data were checked by a double entry in Excel, and SPSS 28.0 was used for statistical analysis). The statistical descriptive measurement data obeyed the normal distribution and were described by the mean ± standard deviation, and the nonnormal distribution was described by the median (quartile). The count data are described by frequency and composition ratio. Paired samples *t* test was used for intragroup comparison, and the 2 independent samples *t* test was used for intergroup comparison. It was found that there was no significant difference in The Chinese Female Sexual Life Quality Questionnaire scores between the 2 groups before intervention (*P* > .05). After the intervention, the sexual desire, vaginal lubrication, sexual arousal, sexual life satisfaction, orgasm, and dyspareunia in the 2 groups were improved; the scores of the intervention group were higher than those of the control group; and the difference was statistically significant (*P* < .05).

There was no significant difference in the scores of HADS and anxiety and depression subscale scores between the 2 groups before intervention (*P* > .05). After the intervention, the HADS score and anxiety and depression subscale scores of the 2 groups of patients were all lower than those before the intervention, the intervention group was lower than the control group, and the difference was statistically significant (*P* < .05; Fig. 3).

## 4. Discussion

PFD threatens the physical and mental health of the majority of women, reduces their quality of life, and the inconvenience and unspeakable embarrassment caused by normal social activities form a shadow in the patient’s psyche, resulting in a loss of confidence in their own value and eventually causing serious psychosocial problems such as isolation and depression.^[9]^ The reasons for this may be due to the elevated progesterone levels during pregnancy, the gravitational effect of the enlarged uterus, the fetus and its appendages, and the change in the direction of pelvic and abdominal pressure causing a direct combined force of the pelvic and abdominal organs pointing to the pelvic floor muscles, combined with the extreme dilatation of the soft birth canal and its surrounding pelvic floor tissues during labor, the elongation or tearing of the muscle fibers, and especially the muscle and nerve damage caused by assisted labor and delivery.^[10]^ This causes varying degrees of pelvic floor function impairment and weakening of pelvic floor muscle support tissues, resulting in decreased postpartum muscle strength, and decreased vaginal tension and contraction, affecting sexual life and even urinary incontinence.^[11]^ Obstetrics and gynecology and related workers are trying to find good methods to improve pelvic floor muscle strength.^[12]^ Among them, postpartum pelvic floor functional exercise is currently a relatively easy and feasible method. It can improve the blood circulation of pelvic floor muscles, promote the recovery of nerve cell function and pelvic floor muscle strength, increase neuromuscular excitability, and awaken some of the nerve cells whose function is suspended due to pressure.^[13]^ This enhances their urinary control ability and vaginal tightness, reduces the incidence of urinary incontinence, and improves the quality of sexual life.

Postpartum pelvic floor rehabilitation is important for the prevention of PFD. Pelvic floor muscle training targets women with reduced vaginal tightness, stress incontinence, and uterine and rectal prolapse after delivery, improves pelvic floor muscle strength, and promotes recovery of pelvic floor muscle function through patient-initiated contraction of pelvic floor muscles and stimulation of muscle nerves.^[2]^ However, most mothers do not know enough about pelvic floor muscle training after delivery and cannot grasp the key points of exercise well enough to achieve the purpose of effective exercise, and Kegel pelvic floor rehabilitation training is influenced by the mothers themselves and their environment, such as education level, disease awareness, family environment, etc, so the rehabilitation effect varies greatly, so the joint targeted, comprehensive, and systematic Greene health education model is of great significance.^[14]^ The Greene health education model is a widely used disease health education promotion model in recent years, the main purpose of which is to improve the public’s health cognition and develop a targeted health education plan so that health education can be implemented in practice.^[15]^ Patients undergo pelvic floor muscle rehabilitation training under the guidance of health care professionals and receive targeted health education, which is conducive to improving self-management awareness, knowledge, and sense of belief, increasing compliance and motivation for rehabilitation training, and improving quality of life.^[16]^ Currently, prevention and interventions framed by the Greene model have been applied to patients with diabetes, esophageal cancer, schizophrenia, and percutaneous coronary interventions, but its research in postpartum pelvic floor functional training is lacking.^[17]^ Therefore, this study used health education combined with biofeedback electrical stimulation in patients to implement an intervention to observe its effect on the recovery of postpartum pelvic floor function.

The pelvic floor muscles play an important role in supporting the pelvic organs, and the number of muscle fibers and muscle strength affect the contraction ability of the anal raphe, so class I and class II muscle fibers are often used to evaluate the postpartum pelvic floor muscle tightness in women, and the decreased quality of postpartum sexual life and stress urinary incontinence is related to pelvic floor muscle relaxation, which can cause vaginal laxity and external urethral sphincter closure dysfunction.^[18]^ Kegel pelvic floor rehabilitation exercises active contraction and relaxation of pelvic floor muscle groups through rhythmic contraction of muscles, thereby awakening the active contraction ability of pelvic floor muscles and thus improving the control of pelvic floor muscles in postpartum women.^[19]^ Greene health education combined with Kegel pelvic floor rehabilitation training can effectively improve the exercise effect on patients.^[20]^ The reason for this may be that by popularizing the knowledge of pelvic floor muscle function in the early postpartum period and providing effective training guidance by medical staff at the time of exposure to Kegel pelvic floor rehabilitation training, patients can better master Kegel pelvic floor rehabilitation training methods, develop individualized training programs, target their training, and actively communicate to establish an immediate feedback mechanism.^[21]^ It not only improves the patient’s self-management ability but also effectively improves the patient’s compliance, develops exercise habits, and maximizes the training effect, thus effectively improving the patient’s postpartum pelvic floor muscle strength, significantly improving the symptoms of PFD caused by pelvic floor muscle relaxation, enhancing vaginal tightness, and restoring its control over the external urethral sphincter and urination.^[22]^

Our study has some innovations and some limitations. Our study was limited by the time factor, the intervention duration was only 4 weeks, and the follow-up and evaluation of the long-term intervention effects were lacking. Our study only investigated the effect of health education combined with biofeedback electrical stimulation on the rehabilitation of patients with postpartum PFD and failed to comprehensively assess the reliability and efficacy of the intervention for patients with postpartum PFD, as well as failed to thoroughly study and follow up the rehabilitation of patients with postpartum PFD after the intervention for a long time. In the future, we plan to conduct a multicenter study in conjunction with other centers to expand the sample size and increase the follow-up time to comprehensively evaluate the reliability and efficacy of this intervention in postpartum patients with PFD.

In conclusion, health education combined with biofeedback stimulation can effectively improve the quality of patients’ sexual lives, improve the pelvic floor muscle strength of patients with postpartum PFD, enhance patients’ confidence, reduce patients’ anxiety and depression, effectively improve patients’ psychological status, and enable patients to resume normal work and life as soon as possible.

## Author contributions

**Conceptualization:** Feng Yuan, Ying Hu, Chunrong Yang

**Data curation:** Feng Yuan, Ying Hu, Chunrong Yang

**Formal analysis:** Feng Yuan

**Investigation:** Feng Yuan, Ying Hu, Chunrong Yang

**Methodology:** Feng Yuan, Ying Hu, Chunrong Yang

**Writing – original draft:** Feng Yuan

**Writing – review & editing:** Feng Yuan, Chunrong Yang

**Supervision:** Ying Hu, Chunrong Yang
